# Affording Sustainability: Adopting a Theory of Affordances as a Guiding Heuristic for Environmental Policy

**DOI:** 10.3389/fpsyg.2017.01974

**Published:** 2017-11-10

**Authors:** Roope O. Kaaronen

**Affiliations:** Environmental Policy Research Group, Department of Social Research, University of Helsinki, Helsinki, Finland

**Keywords:** ecological psychology, affordance theory, pro-environmental behavior, attitude–action gap, environmental policy, socio–ecological systems, nudging, radical embodied cognitive science

## Abstract

Human behavior is an underlying cause for many of the ecological crises faced in the 21st century, and there is no escaping from the fact that widespread behavior change is necessary for socio-ecological systems to take a sustainable turn. Whilst making people and communities behave sustainably is a fundamental objective for environmental policy, behavior change interventions and policies are often implemented from a very limited non-systemic perspective. Environmental policy-makers and psychologists alike often reduce cognition ‘to the brain,’ focusing only to a minor extent on how everyday environments systemically afford pro-environmental behavior. Symptomatic of this are the widely prevalent attitude–action, value–action or knowledge–action gaps, understood in this paper as the gulfs lying between sustainable thinking and behavior due to lack of affordances. I suggest that by adopting a theory of affordances as a guiding heuristic, environmental policy-makers are better equipped to promote policies that translate sustainable thinking into sustainable behavior, often self-reinforcingly, and have better conceptual tools to nudge our socio–ecological system toward a sustainable turn. Affordance theory, which studies the relations between abilities to perceive and act and environmental features, is shown to provide a systemic framework for analyzing environmental policies and the ecology of human behavior. This facilitates the location and activation of leverage points for systemic policy interventions, which can help socio–ecological systems to learn to adapt to more sustainable habits. Affordance theory is presented to be applicable and pertinent to technically all nested levels of socio–ecological systems from the studies of sustainable objects and households to sustainable urban environments, making it an immensely versatile conceptual policy tool. Finally, affordance theory is also discussed from a participatory perspective. Increasing the fit between local thinking and external behavior possibilities entails a deep understanding of tacit and explicit attitudes, values, knowledge as well as physical and social environments, best gained via inclusive and polycentric policy approaches.

## Introduction

Human behavior is a common determinant underlying most of the major ecological crises of the 21st century, and there is simply no escaping from the fact that behavior needs to be changed for socio-ecological systems to take a sustainable turn (Steg and Vlek, 2009; Antal and Hukkinen, 2010). Yet whilst making people and communities behave pro-environmentally is one of the fundamental targets of environmental policy, this foundation is surprisingly often left unspoken, or at least understood from a very limited, non-systemic, perspective. The aim of the present article is to elaborate pro-environmental behavior change policy and intervention analysis by introducing a theory of affordances to the environmental policy community. Affordance theory, which interprets environmental behavior from a dynamical and coupled *systems* or *ecological* approach (Gibson, 1979), is shown to be a promising heuristic for systemic behavior analysis. Particularly, it can help policy-makers locate and make use of ‘leverage points,’ or places where small changes can lead to large shifts in a system’s behavior, for systemic behavior change interventions (Meadows, 1997, 2008; Lockton, 2012). This can help not only individuals, but whole socio–ecological systems to learn to adapt to more sustainable habits.

Affordances are defined in this paper as the ‘relations between abilities to perceive and act and features of the environment’ (Chemero, 2009, p. 150). As Guagnano et al. (1995) and Jackson (2005) note, such integrative approaches, which take into account the dynamical relations between ‘internal’ and ‘external’^1^ behavior antecedents, have traditionally been lacking. I argue therefore that a theory of affordances has a particularly valuable niche to occupy within the multidisciplinary field of environmental policy, since it effectively crosses the artificial divide between internal and external behavior antecedents and studies the dynamical and coupled systems relations between human actors and their (physical and socio-cultural) environment. Moreover, affordance theory invites us to study how this behavior system, as a whole, ‘unfolds over time’ (Chemero, 2013, p. 149). This, in turn, accounts for a more complete picture of environmental behavior and helps us understand why pro-environmental knowledge, values or attitudes are not alone sufficient to induce behavior change (the attitude–action gap), or why everyday environments fail to make the full use of our internal behavior potential. This is particularly relevant since the overwhelming consensus is that despite many or even most of us having pro-environmental attitudes, we are not behaving sustainably (Blake, 1999; Kollmuss and Agyeman, 2002; Abrahamse et al., 2005; Kennedy et al., 2009; Steg and Vlek, 2009).

In the present text, these mismatches between internal and external behavior antecedents are analyzed in terms of affordances, and it is suggested that by increasing pro-environmental affordances we can facilitate systemic and even self-reinforcing pro-environmental behavior change. Moreover, it is also argued below that an affordance-based approach to behavior change intervention can make the best use of the pre-existing latent pro-environmental behavior potential of both humans (capabilities to act, including attitudes, values, knowledge etc.) and everyday environments. This seems to call for a thorough understanding of latent behavior potentials of local populations, suggesting that affordance-based governance should be polycentric (or decentralized), inclusive and participatory, reducing local helplessness and increasing social acceptability.

The crux of this article is therefore to make a case for adopting a theory of affordances as a guiding heuristic for environmental policy. With a heuristic, I mean a fast instrumental and conceptual tool which facilitates ‘exploring and conceptualizing’ pro-environmental behavior, also helping us to ‘identify points of policy intervention’ (Jackson, 2005, vi). A successful heuristic facilitates quick decision-making and helps avoid costly errors. By adopting a theory of affordances as a guiding heuristic, I argue that policy-makers and scholars are better equipped to systemically analyze the ecology of pro-environmental behavior, understand the dynamics between its internal and external antecedents, as well as design appropriate policy interventions.

Adopting the definition from Steg and Vlek (2009, p. 309), environmental behavior is defined in this paper as ‘all types of behavior that change the availability of materials or energy from the environment or alter the structure and dynamics of ecosystems or the biosphere.’ Pro-environmental behavior (abbreviated hereafter as PEB), correspondingly, ‘refers to behavior that harms the environment as little as possible, or even benefits the environment’ (ibid.). The question of what exactly counts as pro-environmental and what does not is not problematized further within the scope of this paper. However, it is worth emphasizing that harming the environment as ‘little as possible’ is not necessarily *pro-*environmental and that pro-environmental behavior in one domain or context might emerge as unsustainable in another.

The body of this article is divided into three main sections. Firstly, in section “The Attitude–Action Gap, or Why We Don’t ‘Walk the Talk”’ the attitude–action gap and its relevance to environmental policy is briefly discussed. Particularly, I suggest that, all too often, pro-environmental behavior research has limited its focal variables to either internal (e.g., values, attitudes, personal norms, habits, and knowledge) or external (e.g., physical infrastructure, economic factors, and institutions) ones (see Jackson, 2005 for an overview). I argue that to overcome the barriers between pro-environmental motivations and behavior, we must understand how our everyday environments provide or constrain the actualization of our pro-environmental internal factors. This requires the simultaneous and dynamical inspection of both internal and external behavior antecedents, as well as particular focus on how these dynamics evolve over time (Chemero, 2013). In section “A Theory of Affordances,” drawing particularly on ecological psychology (e.g., Gibson, 1979) and recent advances in radical embodied cognitive science (e.g., Chemero, 2009), I argue that a theory of affordances provides an effective heuristic framework for studying these coupled and dynamical human–environment behavior systems. In section “Affording Sustainability” I discuss the policy-relevance and potential applications of affordance theory, where affordances can be utilized as leverage points to induce systemic behavior change. In section “Affording Sustainability” I also include a brief meta-empirical survey of how affordance-like ideas have been implemented in environmental policy and psychology, and discuss how intentional adoption of a theory of affordances can hasten the arrival at well-functioning policies at various nested systemic levels. Section “Conclusion” concludes the article.

## The Attitude–Action Gap, or Why We Don’t ‘Walk the Talk’

It is widely accepted amongst those studying pro-environmental behavior (PEB) that a significant gap lies between possessed values, knowledge and attitudes and behavior (Blake, 1999; Kollmuss and Agyeman, 2002; Abrahamse et al., 2005; Jackson, 2005; Kennedy et al., 2009; Steg and Vlek, 2009). In other words, an attitude–action gap, a knowledge–action gap or a value–action gap exists between internal human factors and behavior patterns. For practical purposes, I from here on refer to this discrepancy between internal factors (such as attitudes, values, knowledge, personal norms, intentions and emotions) and behavior simply as the attitude–action gap, humbly acknowledging that this does a disservice to the great body of research focused on studying the relationships between these individual internal factors and pro-environmental behavior (see Kollmuss and Agyeman, 2002; Abrahamse et al., 2005; Steg and Vlek, 2009 for an overview on the topic).

The attitude–action gap does not imply that internal factors do not have *any* effect on pro-environmental behavior, but rather that a great amount of PEB cannot be explained with internal factors alone. Generally, it seems that internal factors are more likely to lead to change in low-cost (low in time and effort) actions than in high-cost behavior (Kollmuss and Agyeman, 2002; Abrahamse et al., 2005; Steg and Vlek, 2009), although not all research fully supports this (e.g., Hunecke et al., 2001). Moreover, the case seems to be that internal factors seem to correlate more strongly with behavior when they are specific to a certain domain. This is, perhaps, common sense: positive recycling attitudes strongly predict recycling behavior (and not, for example, travel behavior), whilst more generic pro-environmental values do so only to a much lesser extent (Vining and Ebreo, 1992).

The attitude–action gap is, at the root of it, rather intuitive. Many people with pro-environmental intentions will have experienced the uncomfortable feeling of cognitive dissonance when they have taken part in environmentally harmful yet seemingly banal activities such as air travel. Simply, as Vining and Ebreo (1992, p.1604) observe, ‘it is easier to be concerned about the environment than it is to act on one’s convictions.’ This mundane and banal phenomenon, however, takes on direct policy relevance when combined with an urgent need for humans to change their behavior patterns and habits to tackle ongoing ecological crises. We talk the talk, but systemically fail at ‘walking the talk’ (Kennedy et al., 2009). Since significant portions of national populations are pro-environmentally motivated, translating these latent pro-environmental behavior potentials into action becomes an imperative task for environmental policy. For one example, Kennedy et al. (2009) found that Canadians adhere much more strongly to the ‘New Ecological Paradigm’ world-view (which states, inter alia, that ‘humans and other species are intricately connected’) than to the so-called ‘Dominant Social Paradigm’ (‘mankind was created to reign over the earth’).

A comprehensive literature review on the attitude–action gap is beyond the scope of this article. Fortunately, such work has already been done, notably by Kollmuss and Agyeman (2002), Abrahamse et al. (2005), Jackson (2005), and Steg and Vlek (2009). Briefly, however, it should be noted that studies on the relations between mental models and behavior have progressed significantly from the oldest and simplest models known as ‘rational,’ ‘linear,’ or ‘information-deficit’ models, which established a direct linear relation between knowledge, values and behavior. More complex and nuanced models have taken into account how attitudes, norms, beliefs, intentions, emotions, affect, altruism, locus of control, self-identity and a large variety of other variables influence pro-environmental behavior, also including sociological factors, situational variables and, to a somewhat limited extent, the structural and physical environment (Steg and Vlek, 2009, p. 314).

I argue, however, that many of these accounts on pro-environmental behavior – important as they are for understanding the complexities of human practices – suffer from a very fundamental a priori assumption, which limit cognition ‘to the brain’ (Rockwell, 2005). In this paradigm, often implicit in environmental psychology, contextual factors, if considered at all, have usually been ‘introduced in the form of subjectively perceived environment,’ and not as systemic ecological situations (Hunecke et al., 2001). Moreover, when the effects of external (such as economic) factors on behavior have been studied, it has often been done so with the cost of excluding internal human factors. Integrative approaches, which take to account the dynamical coupling between internal and external behavior variables, have traditionally been scarce (however, see Guagnano et al., 1995; Stern, 2000; Hunecke et al., 2001; Jackson, 2005).

This is, of course, traceable to a long tradition of Cartesian materialistic thinking, often implicit in the psychological and cognitive sciences (Heft, 2001; Rockwell, 2005; Chemero, 2013; Ch. 7 in Reed, 1996). That is, the notion that behavior and cognition are *ecological*, construed dynamically in an ecological system, is most often downplayed in favor of more limited approaches which reduce cognition and behavior to the internal domain (e.g., mental representations). Whilst this might be a pragmatic and even useful limitation at times, treating human cognition as a ‘static’ entity, ontologically separable from outside variables, can also be wildly misleading (Kurz, 2002, p. 269). What is suggested below is that rather than focusing on single static variables underlying behavior we should take a dynamical stance, such as that provided by a theory of affordances.

Affordance theory originates from the field of empirical and theoretical research known as *ecological psychology*. Ecological psychology draws mainly from perceptual psychologist James J. Gibson’s work (most influentially Gibson, 1979), which emphasizes the dynamical and systemic coupled relations between animals and their physical environment. As used in this text, ecological psychology should not be confused with environmental psychology or other strains of research going by the name of ecological psychology (such as Roger Barker’s), although many similarities between these fields exist (see Heft, 2001 for a useful overview). I argue that ecological psychology and its more recent descendants in radical embodied cognition theories (e.g., Chemero, 2003, 2009, 2013 as well as Rockwell, 2005) should be revisited in order to understand more comprehensively the role our everyday and urban environments play in shaping our environmental behavior. This is elaborated in detail in sections “A Theory of Affordances” and “Affording Sustainability” in the form of a theory of affordances.

A few caveats are in place before moving onward. I am not proposing a silver bullet to solve the problem of the attitude–action gap altogether. As Kollmuss and Agyeman (2002, p. 248) rightly note, the gap is ‘such a complex one that it cannot be visualized in one single framework or diagram.’ This is wholly unsurprising from a systems theoretical point of view, where it is generally understood that no static conceptualization or model can capture the whole complexity of a contingent system (Meadows, 2008). Indeed, to map the complete causality underlying a behavior system is practically impossible, since it would take an astronomical scale (Rockwell, 2005). Accordingly, ‘there will always be something of a tension between simplicity and complexity’ in modeling behavior, and a ‘good conceptual model requires a balance between parsimony and explanatory completeness’ (Jackson, 2005, p. 23, vi). Therefore, what is merely suggested below is that a theory of affordances provides us with a pragmatic (see Rockwell, 2005) and adaptable heuristic for understanding and intervening with behavior from a systems perspective. Such a heuristic not only facilitates and hastens the arrival at working policy solutions, but also importantly helps us avoid unintended consequences and making costly mistakes. For now, in section “A Theory of Affordances,” however, it is in place to provide a more detailed description of what exactly we mean when talking about a theory of affordances.

## A Theory of Affordances

An affordance, in its simplest – yet most philosophically impoverished – definition, refers to the action possibilities provided by objects or environments. Whilst, as is elaborated below, the concept is in fact significantly more nuanced than this, the aforementioned definition of affordances has been widely adapted by, for instance, the design community: ‘when used in this sense, the term *affordance* refers to the perceived and actual properties of the thing, primarily those fundamental properties that determine just how the thing could be possibly used’ (Norman, 2002, p. 9). It follows then, that a chair provides support and thus *affords*^2^ humans with (or ‘is for’) sitting. Apples afford, among a huge variety of behavior, throwing, eating, baking and cutting. Bananas afford – explaining their huge urban popularity – easy, fast and locally clean eating as well as exportability, since they ripen after picking.

However, a more nuanced treatment of affordances does not consider affordances as *properties* of objects or environments, but rather in terms of ecological situations. As Chemero (2003, 2009) remarks, affordances are functionally meaningful features of whole situations. These whole situations are better defined as fluctuating behavioral fields emerging from brain–body–world interaction (Rockwell, 2005, 2010). Here, the similarity to Lewin’s (1951) field theory, a theory positing human behavior as “a function of a dynamical ‘field’ of internal and external influences,” is obvious (Jackson, 2005, p. 26; see also Heft, 2001). Affordances from this more refined perspective are not – contra the popular understanding (see e.g., Kurz, 2002; Norman, 2002) – dispositional properties of things or environments, but rather functionally meaningful ‘relations between abilities to perceive and act and features of the environment’ (Chemero, 2009, p. 150; c.f. Turvey, 1992). The environment here is to be understood to refer to the whole of the material world, physical, cultural and social environments included. This is the definition of affordances used in the remainder of this text.

Affordances are therefore dynamical and coupled organism–environment relations, hence through and through systemic and ecological (Chemero, 2009). A chair, given the right conditions, affords sitting for an erect bipedal species such as ours, whilst its affordances are wholly different for other species not adapted to walk and sit. Affordances are therefore not ‘psychologies of things’ (contra Norman, 2002), but rather psychologies of organism–environment relations. Affordance theory posits that active cognitive agents perceive and experience the world in terms of affordances, or functionally meaningful relations with the environment. We do not perceive the world passively as having pre-given objective and action-neutral properties, but rather as active opportunities for action (Ramstead et al., 2016). Our everyday lives are ridden with affordances, and they are continuous, dynamic, reciprocal and evolutionary processes: affordance-sets constantly affect organisms and populations, whilst organisms continuously adapt to and modulate the niches (or sets of affordances) they inhabit (Heft, 2001, xxix; Chemero, 2003, p. 190; Reed, 1996, p. 26).

The ontology of affordances is therefore one which attempts to effectively cross the artificial subject–object divide (Chemero, 2009).^3^ This is perhaps best captured by the following oft-cited, yet slightly cryptic, quote by Gibson (1979, p. 129):

‘an affordance is neither an objective property nor a subjective property; or both if you like. An affordance cuts across the dichotomy of subjective-objective and helps us to understand its inadequacy. It is equally a fact of the environment and a fact of behavior. It is both physical and psychical, yet neither. An affordance points bothways, to the environment and to the observer.’

Whilst this might seem unnecessarily muddling and counterintuitive to some – or even violative toward the law of non-contradiction (*viz.* ‘neither an objective property nor a subjective property’ or ‘both if you like’) – Gibson’s definition contains a valuable insight: when understood ecologically, behavior is not constrained to the perceiver nor to the perceived, but is rather a dynamical and coupled systems relation between the perceiving organism and the environment it inhabits. From this perspective, it is distasteful to reduce cognitive systems to the brain (or even body), but cognition and behavior rather emerge over time from a ‘dynamical brain–body–world nexus’ (Rockwell, 2005; Anderson et al., 2012; Hutto and Myin, 2012). Hence affordances imply a degree of extended cognition: the perceived world is not construed by the brain or mind, but rather emerges from the interaction between a nervous system, a body capable of perceiving and an environment which affords perception (via, for example, latent information in the form of structured ambient light in the environment) (Gibson, 1979; Reed, 1996; Chemero, 2003, 2009; Rockwell, 2005, 2010; Anderson et al., 2012). Affordance theory implies that meaning is not construed by the brain alone (by any means of ‘mental gymnastics’), nor is it merely a social construct, but rather is latent in the environment and (directly) perceivable in organism–environment interactions (see the ‘radical embodied cognitive science’ of Chemero, 2003, 2009). In contrast to inferential theories of perception, where ‘meanings arise inside animals, based on their interactions with the physical environment,’ affordance theory suggests that ‘the animal simply gathers information from a meaning-laden environment’ to actualize some function (Chemero, 2003, p. 181; see also Gibson, 1979, p. 238–263). Meaning, cognition, perception, and thus also behavior, are thoroughly ecological. Hence, of course, *ecological* psychology.

Importantly, as Gibson’s quote above implies, affordances are not idealistic (in the ontological sense), and despite their hardly tangible nature, affordances are indeed real, perceivable and empirically observable (Chemero, 2003, 2009; Heft, 2003; c.f. Kurz, 2002 who, among others, claims affordances are mere subjective perceptions). Chemero (2003, p. 187) suggests that for us to understand affordances we should consider the ‘taller-than’ in the statement ‘Shaquille is taller than Tony.’ The taller-than is neither a property of Shaquille or Tony, yet it is still an empirically observable and real relation in the whole situation. Affordances are equally real. To further elaborate, Chemero (2009, p. 150), drawing on Dennett (1998), likens affordances to the state of being ‘lovely’: a hippopotamus can continue to have the potential for being lovely even when it is not, at that moment, observed by another organism. In other words, the hippopotamus’ physical structure has latent *potential* to be lovely for a *potential* observer, even if the affordance of ‘being lovely’ is only actualized when complemented by another organism which has the abilities to perceive and experience its latent loveliness, given that the right conditions are met. Affordances are, as Chemero (2003, p. 193) notes with dry wit, ‘lovely.’

Our everyday lives make use of innumerable affordances even when we are not conscious of them (I would argue that we mostly are not), and affordances do not require us to be able to consciously locate them. Take for instance Polanyi’s (1958/1974, 1966/2009) well-known example that we can, without effort, recognize familiar faces without being able to explicate how we achieve this (i.e., familiar faces afford recognition).^4^ A similar tacit use of affordances is illustrated by the so-called ‘gaze heuristic,’ by which humans (and, it seems, dogs) can catch flying objects unconsciously (and without any mental gymnastics such as trajectory calculation) by simply fixing their gaze on the object, starting to run, and adjusting running speed so that the angle of the gaze remains constant (see the ‘ecological rationalism’ of Todd and Gigerenzer, 2012). The ‘catchability’ affordance of an object is, therefore, specifically an active organism–environment relation. Moreover, to tacitly recognize affordances is no trick unique to humans; all organisms are evolutionarily adapted to their ecological niche and can make sense of the affordances within it. Charles Darwin, who made less famous advances in animal perception, noted how earthworms very delicately adapt to the affordances within their ecological niche (see Reed, 1996, p. 20–28; Darwin did not, of course, use the term ‘affordance’). We do not need to consciously recognize affordances to make use of them – certainly earthworms do not, at least not to our human standards of consciousness. However, we *can* knowingly identify and recognize affordances sufficiently for us to modulate them, as will be discussed in section “Affording Sustainability.”

**FIGURE 1:**
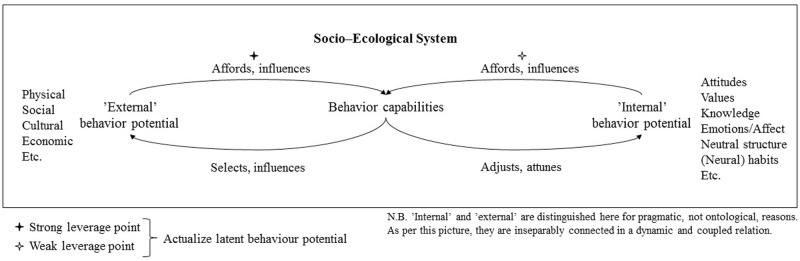
The dynamical and ecological behavior system.

In this section I have asserted that organism–environment relations are coupled and dynamical systems. For our purposes, this means that human cognition and behavior are, on an ontological level, formed simultaneously, continuously and dynamically from both internal (organismic) and external (environmental) behavior potential. Moreover, affordance theory implies that we not only study the way external and internal factors cause changes in behavior, but rather ‘the way the system as a whole unfolds over time’ (Chemero, 2013, p. 149). **Figure 1** illustrates this as a coupled and dynamical feedback system. The rationale for modeling environmentally significant behavior in such a non-linear fashion, with potential for positive feedback (this is returned to in the following section), stems from affordance theory’s recent resurgence in radical embodied cognitive science, which attempts to describe psychology by combining ‘non-linear dynamical modeling with ideas about the nature of the mind’ (Chemero, 2013, p. 145; see also Rockwell, 2005 and Chemero, 2009). This is necessary since, as Chemero (2013, p. 148) continues, it ‘is only for convenience (and from habit) that we think of the organism and environment as separate; in fact, they are best thought of as forming just one non-decomposable system.’

Here we can identify a processual scheme dynamically interconnecting ‘internal factors,’ ‘behavior capabilities’ (e.g., socio–physical abilities to act) and ‘external factors.’ Importantly, this behavioral process is not linear, but all events (arrows) of the process are interconnected and active simultaneously and constantly. To paraphrase Gibson (1979, p. 240), behavior is a flux and not a sequence – a continuous evolutionary act which is ceaseless and unbroken. Behavioral systems are ‘processual’ and not discrete (in terms of process philosopher Rescher, 1996, 2000) or ‘loopy,’ and not ‘linear’ (as per enactivists Hutto and Myin, 2012: 6 or Varela et al., 1991). The ecological behavior system is dynamical (it evolves continuously) and coupled (its constitutive parts are interconnected, and a change in one variable results in changes in the others). This situation model in **Figure 1** represents, essentially, a self-organizing coupled dynamical system where ‘the river molds the banks and the banks guide the river’ (Bateson, 2000, p. 83). Whilst **Figure 1** presents internal and external factors as collections of variables, we could also choose this model to analyze dynamics between specific internal and external variables. Note also that whilst affordances are generally taken to refer to merely the arrow connecting external factors to abilities, I have also chosen to use the verb ‘afford’ to connect internal behavior potential with abilities. After all, the latent structure of internal factors affords individuals with behavioral abilities, even if not always to the same force as the structure of external factors. This figure is returned to with practical examples in the following sections.

I argue below that the notion that our everyday worlds are infused, often unknowingly to us, with innumerable affordances, takes on a very political nature. Whilst affordance theory is generally considered a realistic or naturalistic description of organism–environment relations, it can, and arguably should, also be politicized. What kinds of affordances do we reinforce, foster and inhibit, and how is this reflected in everyday behavior patterns? More precisely, how (if at all) do the most prevalent features in our socio–ecological system afford pro-environmental behavior, and are available affordances equal for different populations? Affordance theory presents us a framework for studying the ecology of human behavior, and particularly for focusing on how our everyday and urban environments systemically nudge individuals and local populations to behave in environmentally significant patterns and habits. A better understanding of local behavior potentials (internal and external) and their dynamics over time can facilitate the design of urban and everyday environments which help to actualize these potentials, resulting at best in self-reinforcing systemic learning patterns. This would suggest for local, decentralized (or polycentric) and even participatory governance, where policy-designers are more specifically attuned to local behavior potentials and capabilities. An imperative question arises here for those involved with environmental policy. How do we, as a society and culture, as individuals, as local communities, as policy-makers, *afford* sustainability? These issues are elaborated in the following sections.

## Affording Sustainability

Having outlined the conceptual aspects of a theory of affordances, it is now time to consider its policy-relevance. I have suggested above that environmental policy-makers should adopt affordance theory as a guiding heuristic for policy development, particularly to understand the attitude–action gap in environmental behavior and target policy interventions to induce systemic behavior change. An efficient and coherent heuristic is more than a semantic advantage; a good heuristic model can shape the way in which we intuitively perceive the world and therefore promptly aid policy- and decision-makers in identifying points for policy intervention, hastening the arrival at working-as-intended policies and helping to avoid costly (in time, effort and money) mistakes (Jackson, 2005).

Since affordance theory provides us with a through-and-through systemic understanding of environmentally significant behavior, focusing on the dynamics between internal and external behavior antecedents, it helps us locate systemic leverage points (Meadows, 1997, 2008; Lockton, 2012) for policy intervention. Leverage points are here to be understood as ‘places in the system where a small change could lead to large shift’ in the system’s behavior (Meadows, 2008, p. 146). Since environmental behavior is a coupled and dynamical system which evolves over time (see **Figure 1**), by making use of leverage points policy-makers have the capacity to help this system learn to behave more sustainably. This is in Bateson’s (2000) terms *deutero-learning* (learning to learn), in other words inducing second-order change to the system to complement the usual first-order trial and error (environmental behavior as usual). By understanding and leveraging these feedback loops, we can, again quoting Bateson (2000, p. 274), ‘not only solve particular problems but also form *habits* which we apply to the solution of *classes* of problems.’ A central task for environmental policy-makers and scholars is therefore to help our socio–ecological system – not just its individual constituents – to learn to behave more sustainably. What follows is an attempt to describe such systemic learning.

To understand the attitude–action gap in terms of a theory of affordances, we should begin with asking why our everyday niches *do not* afford sustainable behavior. Here Norman’s (2002) insights from cognitive science and design are of direct relevance for environmental policy. Norman (2002, p. 51) suggests that two ‘Gulfs’ separate internal mental states from being complemented by external physical ones, namely the Gulf of Execution and the Gulf of Evaluation.

The first of these gulfs is the Gulf of Execution, which exists when the actions provided by a system do not match those intended by a person, or when a system does not allow a person to execute the intended actions directly and without significant effort (Norman, 2002). In the case of the attitude–action gap then, this would equal to a person with high pro-environmental intentions (let us signify this here with INT+, for internal factors) yet with low action possibilities provided by their ecological niche (EXT-, for external factors).

The second gulf Norman (2002, p. 51) specifies is the Gulf of Evaluation. The Gulf of Evaluation exists when a system does not provide physical representations that can be directly perceived and interpreted in terms of intentions and expectations of a perceiver. In other words, the Gulf of Evaluation exists during lack of functionally meaningful feedback. For this Gulf to be crossed, the amount of effort that a person must exert to ‘interpret the physical state of the system’ must be low and the person must be able to determine how well their expectations and intentions are met. Systems should provide information that is easy to acquire and interpret, and match the way in which the person perceives the system. Because ‘people generally do not know which and whose behaviors significantly affect resource use,’ or at least such knowledge is bound to be vague and filled with misunderstandings, feedback is important from an educational point of view, giving instructions for future behavior (Steg and Vlek, 2009, p. 310). A system intended to overcome the attitude–action gap must therefore not only provide simple and comparative feedback, but also provide functionally meaningful ‘feedforward’ (‘how to act from here on’) (Lockton, 2012).

Consider now, drawing back on **Figure 1**, the attitude–action gap in terms of dynamical and coupled human–environment relations, or affordances. If a population’s pro-environmental internal set (values, knowledge, attitudes etc.) is high (INT+) and we are witnessing a lack of behavior, the heuristic answer per a theory of affordances would be that the niche does not provide sufficient affordances for the actualization of the internal sustainability potential (thus EXT-). Most likely, this is due to insufficient action possibilities (Gulf of Execution) and feedback (Gulf of Evaluation).

Now, consider that by policy means we cross the Gulfs of Execution (make the system afford physical actions) and Evaluation (make the system provide feedback/feedforward). In other words, we alter our niche to have better capacities for actualizing our latent pro-environmental potential, thus increasing sustainable affordances. This would particularly entail intervening with the strong leverage point in **Figure 1** (‘External’ behavior potential → Behavior capabilities, or altering the material aspects of the environment). Now we have a coupled feedback loop of INT+ and EXT+. With the increase of affordances in our niche (via EXT+), the latent potential of INT+ can be actualized. That is not to say that intervening with the weak leverage point (see **Figure 1**) is unnecessary here, since abilities to utilize any external factors also have to be taught and learned – the case is merely that without the strong leverage point being activated (e.g., recycling being physically possible) no amount of weak leveraging will suffice.

A case example demonstrating such a positive sustainable feedback loop between internal and external factors would be a couple, call them Alfa and Beta, both possessing high pro-environmental attitudes and knowledge (INT+), and thus high latent potential for recycling, living in a suburban environment without easily accessible recycling systems (EXT- due to a Gulf of Execution; e.g., inconvenient drop-off recycling locations). Note also that their waste disposal system provides no feedback or feedforward as to how they are acting or how they should act (EXT-). To remind Alfa and Beta (say, via information campaigning) about their unsustainable action is unlikely to substantially change behavior, and it might at worst result in Alfa and Beta experiencing cognitive dissonance and thus blocking the dissonant information or delegating responsibility elsewhere (by means of self-justification).^5^ Now, imagine a local environmental policy-maker, after surveying the local populations’ environmental perceptions and identifying latent pro-recycling attitudes, deploys each household in the suburb with easily accessible curbside recycling systems (crossing the Gulf of Execution).

Alfa and Beta now have affordance for recycling (EXT+ and INT+). Moreover, since it is now convenient for them to recycle, the very act of recycling is likely to strengthen their pro-environmental identities and attitudes. One explanatory theory for this is the theory of cognitive dissonance, which suggests that humans have a tendency of adjusting attitudes to conform to behavior patterns (Cooper, 2007). This increase in internal behavior potential makes it possible now for Alfa and Beta to further adjust their ecological niche and fit their everyday environments with less wasteful affordances (e.g., by altering the prevalence of certain products and appliances). This again reinforces their pro-environmental identities, and so on. Whilst the recycling example is a mundane one (and arguably a minor factor in the global ecological crisis), it is one of the more researched fields of PEB and therefore serves the purpose here to illustrate such cyclic systemic learning patterns. Steg and Vlek (2009, p. 312) note, accordingly with the logic of **Figure 1**, that the ‘introduction of recycling facilities may result in more positive attitudes toward recycling (e.g., because it is more convenient), and positive attitudes may in turn result in higher recycling levels.’^6^ Vining and Ebreo (1992, p.1604) research on recycling similarly concludes (inter alia) that increased recycling opportunities (implementation of curbside recycling) not only significantly increased recycling behavior but also led to an increase in positive ‘global environmental’ and ‘specific recycling’ attitudes, thus ‘strengthening already positive environmental attitudes.’ Guagnano et al.’s (1995) study also concluded that having a curbside bin increased pro-environmental recycling behavior (by reducing barriers between latent pro-recycling attitudes and action) and, importantly, awareness of the social and environmental consequences of recycling. Moreover, a similar feedback loop (or ‘positive interactive cycle’) has also been found by Kyttä (2003, p. 98, Kyttä, 2004) in studies on child-environment relationships: a child-friendly environment ‘allows a positive interactive cycle to develop between a child and the environment’ where ‘actualized affordances for their part motivate the child to move around more in the environment, which creates more possibilities for new affordances to become actualized.’

Basically, we have here the potential for systemic leveraging, where by actualizing a sufficient number of pro-environmental affordances (by intervening with the strong and weak leverage points of **Figure 1**) we can reinforce the pro-environmental identities and motivations of populations, which again further spurs PEB and potentially even further spontaneous pro-environmental modulation of everyday environments. In such a case we can imagine the behavior system in **Figure 1** running smoothly, and to an extent self-reinforcingly, evolving over time toward more sustainable habits. For Reed (1996), the whole notion of culture arises from this kind of bootstrapping, where the agglomeration and proliferation of certain types of affordances forms a ‘field of promoted action,’ which spurs new practices, ideas/inventions and socio-cultural interactions. This is also known as the ‘ratchet-effect,’ or the notion that human socio-technological culture accumulates (often irreversible) modifications over time (Tomasello, 1999; Tennie et al., 2009). This ‘cultural ratchet’ of cumulative learning, of course, also involves the social dimensions of teaching, social imitation and norm conforming (Tennie et al., 2009). We are no longer dealing here with individual organism–environment relations, but rather a ‘rich landscape of affordances’ (Rietveld and Kiverstein, 2014) which promotes certain social practices (see Shove et al., 2012) and reinforces what Ramstead et al. (2016) have recently called ‘shared expectations’ or ‘local ontologies’ of a population (behaving in ways which others expect one to behave). These shared expectations and local ontologies are embodied at various levels from brain networks, cultural artifacts and constructed environments, which further reinforce enculturated practices (ibid.).

For instance, when enough people are incentivized to recycle and the built environment supports this behavior (i.e., recycling is systemically afforded), it becomes a normalized social and cultural practice, or a cultural affordance, where we expect others to expect us to recycle (see section “Object-Level Affordances” for a case example). A cultural affordance in this context refers to the possibilities for action which depend on the skillful leveraging of ‘explicit or implicit expectations, norms, conventions, and cooperative social practices’ (see Ramstead et al., 2016, 3; although more specifically, Ramstead et al. call this a ‘conventional’ cultural affordance). The principal lesson here for those involved with environmental policies is therefore that by actualizing, or locating and activating in large enough numbers what I have called systemic leverage points, the recycling case being only one of innumerable possibilities, we not only promote individual sustainable behaviors but also reinforce the emergence of sustainable pro-environmental sociocultural practices and hasten the transition toward a more sustainable culture. This implies that we are essentially helping our socio–ecological system to learn more sustainable habits. A central task for those involved with environmental policies therefore emerges as the need to redesign our ecological ‘niche,’ or ‘designer environment’ (Ramstead et al., 2016), so that its rich landscape of affordances systematically promotes pro-environmental behavior. In such an ecological niche, pro-environmental behavior would emerge in many respects as the path of least resistance and the default form of life.

That is not to say that these positively reinforcing feedback loops would go on forever, since they would eventually settle down to, or oscillate around, some relatively steady state, depending on the availability of affordances, or be disrupted by external forces. Moreover, a single feedback loop might not spill over to other PEB domains (e.g., from recycling to increased bicycling), or at least current research is very dubious as to whether or not this is the case: spillover effects have been reported to be both positive (PEB in one domain leads to a PEB in another) and negative, where, quite concerningly, PEB in one domain rebounds as a lack of PEB in another (Truelove et al., 2014). The case seems to be, though, according to Truelove et al.’s (2014, p. 132) meta-empirical review, that “those who engage in a PEB because their environmental identity has been activated will be likely to exhibit positive spillover because the participants’ role will get reinforced and strengthened as the result of the initial decision.” Contrarily, external coercing of PEB might have a converse effect. This suggests that we should particularly make our everyday environments afford sustainable actions that reinforce *pre-existing* latent pro-environmental internal factors, making us perceive that we are (knowingly and willingly) acting in consonance with our pro-environmental identities and not enforced or coerced by external authorities to do so.^7^ This is a relevant observation for environmental policies, where behavior interventions should particularly be implemented in domains where significant latent pro-environmental behavior potential (e.g., attitudes or knowledge) exist. Here, the provision of material environments which afford PEB has higher potential to lead to spillover effects and positive feedback loops in PEB. Moreover, since pro-environmental internal factors are, in many respects, pre-existing unutilized resources (as exemplified by the attitude–action gap), their actualization is also a cost-effective way of inducing pro-environmental behavior and habits.

Making the best use of affordances as leverage points is a fascinating opportunity for those involved in environmental policy and behavior interventions, although any applications must be preceded by a thorough understanding of system dynamics. Simplistic ‘if-you-build-it-they-will-come’ or ‘one size fits all’ policy approaches are insufficient for identifying leverage points (see Ostrom, 2010 for criticism on such top-down approaches), since affordances are transactional. To make the full use of these self-reinforcing feedback loops and sociocultural ratcheting processes, we need to understand *which* external structures complement a *certain* population’s set of internal factors. This calls for local, decentralized and perhaps even participatory policy approaches, where local behavior potentials (internal and external) are thoroughly charted before the implementation of behavior change strategies. This also a political reasoning for not defining affordances as uniform ‘properties’ of things or environments, since physical environments can afford environmentally significant behavior patterns very unequally. Affordance theory takes on a very political nature here, and must be particularly sensitive toward socioeconomic factors and behavior capabilities. Firstly, individuals might have variety in their ability to utilize affordances and transform resources into valuable activities. Second, the distribution of environmentally significant affordances might be fundamentally unequal between local populations and socio-economic groups (see the ‘capability approach’ of, e.g., Sen, 1995 for similar arguments). For instance, targeting costly information campaigns or ‘blaming strategies’ at non-recycling low-income families might be unfairly patronizing if recycling affordances are scarce to begin with (Jackson, 2005, p. 54). Moreover, ‘fetishizing’ actions such as recycling – to which less fortunate populations might have less affordances – at the expense of letting ‘political minefields’ such as air travel off the hook is certainly questionable on moral and political grounds (see Capstick et al., 2015).

Therefore, affordance theory seems to quite naturally call for polycentric (Ostrom, 2010), local and inclusive governance which understands the behavior potentials (internal and external) of local populations and encourages, facilitates and guides local populations to act accordingly with their latent pro-environmental attitudes. Indeed, participatory problem solving of this kind has also been claimed to reduce helplessness (since it helps people understand and explore problems) and thus induce sustained and long-term pro-environmental behavior (Kaplan, 2000; see Jackson, 2005).

### Applied Affordances

I have stated above that socio–ecological systems, everyday environments included, are thoroughly infused with affordances. To comprehend the full potential of affordances in environmentally significant decision-making, it is worth explicating how diverse the analysis and leveraging of affordances can be. Here, scalability and adaptability are what truly make a theory of affordances stand out from other theoretical models.^8^ Since affordances are systemic relations, an affordance is a scalable heuristic applicable to whatever system we are interested in observing. We can therefore choose to analyze affordances from a nested order of systems (Gibson, 1979, see also Ostrom, 2005). This systemic nature of affordance theory makes it an incredibly versatile analytical tool, basically applicable to any area of interest of environmental policy. Consider, for example, how we could choose to study affordances related to (1) objects and everyday items, (2) households (3) urban environments or (4) socioeconomic systems, and how this can inform us about potential leverage points for environmental policy intervention. These adaptations of affordances are briefly discussed below with affordance-relative case studies.

#### Object-Level Affordances

Physical objects are perhaps the most intuitive of affordance-relatable entities. As was the case in this article, introductions to affordance theory usually begin with imagining what functions objects afford for humans. It comes then as no surprise that affordances of objects have been studied with quite some detail, particularly by the design community. For instance, in recent years several authors have published under the umbrella-term of ‘design for sustainable behavior,’ which (often drawing on the work of Norman) study how objects afford pro-environmental behavior and how variables such as understandability, ease of use and functional meaningfulness affect sustainable product use (see e.g., Lockton et al., 2008; Bhamra et al., 2011; Lockton, 2012; Selvefors, 2017). Often, though, affordances are in this context generally defined merely as properties of objects, a conception against which I have argued in this text (in favor of affordances as systemic animal–environment relations, see section “A Theory of Affordances”).

A great example of objects affording sustainability can be found in the Finnish bottle deposit-refund system, where each bottle or can sold is placed with a deposit ranging from 10 to 40 cents added to the beverage’s retail price (PALPA, 2017). The system gives consumers monetary incentive to recycle, since the deposit is refunded when bottles and cans are returned to stores and kiosks. The bottles afford a visual prompt for recycling (overcoming the Gulf of Evaluation), and recycling points are abundant (crossing the Gulf of Execution, since each store that sells deposit-items is required to also receive them).

Technically speaking, it is not the bottles and cans alone which afford recycling here, but rather both the objects and the whole recycling system they are embedded in. However, it is clear that ‘recycling’ has become a prominent affordance (functional meaning) which consumers perceive when encountering a bottle or can in the Finnish culture. The deposit system has been hugely successful, with the recycling rate of bottles and cans ranging from 89 to 98%. Arguably, a point has also been reached where the recycling system reinforces shared expectations and social practices (in other terms, recycling has become a social or cultural affordance), whereby deviations from this norm are considered unacceptable (circa 90% of the population sample self-reportedly always/often recycle bottles and cans; Blom et al., 2010). However, I suspect the pro-environmental affordance-potential is not used to its full capacity in this case, since feedback from recycling mainly concerns monetary benefits, and to a much lesser extent environmental welfare.

#### Household Affordances

Abrahamse et al. (2007) acknowledge in their study on energy consumption behavior, households are responsible for a highly significant portion of greenhouse gas emissions, and domestic environments should therefore be considered an important target group for behavior change interventions. The authors (Abrahamse et al., 2007, p. 266) note that whilst knowledge itself predicts pro-environmental behavior rather poorly, tailored information and feedback as well as feedforward (in the form of goal setting) can be effective strategies for encouraging energy conservation. This is particularly the case with continuous electronic feedback, made possible by digitalized energy systems. In Abrahamse et al.’s (2007) study, experimental groups were given access to an online website with information on energy consumption and related ecological problems, along with a list of tailored energy-saving measures and an online tool which could calculate relevant and practical energy-saving means. Basically, the tool gave simple and comparative feedback and feedforward on how to reach the intended goal of 5% energy consumption reduction. The 5-month long intervention resulted (among a variety of other interesting findings) in the experimental groups lowering their direct (gas, electricity and fuel) energy consumption by 8.3% as opposed to the control group, whose direct energy consumption increased by 0.4% (although indirect energy consumption was not affected nearly as strongly as direct energy use).

This would suggest, although the authors do not discuss the results in terms of affordances, that when household energy systems are designed to afford sustainable behavior (in this case, by crossing the Gulf of Evaluation), they have the potential to significantly strengthen pro-environmental behavior patterns. Importantly, as opposed to a control group, the intervention also resulted in heightened energy conservation knowledge within the experimental groups, signaling potential for a sustainable feedback loop, where not only heightened explicit knowledge but also tacitly acquired practical ‘know-how’ would further increase the ability to adopt more sustainable consumption habits (see Darby, 2006). Affordance theory also implies that the intervention would have likely been even more effective had the Gulf of Execution and Evaluation been crossed more efficiently: instead of using a website (which must be accessed with significant intent) the information could be ready-to-hand^9^ at a constantly visible location within the household (e.g., an interactive LCD-screen). With the dawn of smart energy systems in digitalized domestic environments, such high pro-environmental affordance systems could become mainstream in the near future. This is potentially a big step forward from current electric billing, which affords sustainable energy consumption behavior particularly poorly due to technical and rare (e.g., quarter-yearly) feedback and lack of prompts regarding how to change behavior.

#### Urban Affordances

Marcus et al. (2016) explicitly discuss affordances in the context of urban design. The authors note that ‘most approaches to sustainable urbanism still share the conception of the humans–environment relations that characterized modernism’ and therefore do not emphasize the dynamical systemic properties of urban environments. Instead, affordances could form the core of a ‘new epistemological framework of the human–environment relation in sustainable urbanism’ (Marcus et al., 2016, Abstract, 440). Against the backdrop of the Cartesian human–environment dualism implicit in much of urban design, we should rather advocate a dynamical and interactive two-way understanding of the relations between humans and the urban environment. Marcus et al. (2016, p. 445), in fact, go as far as recognizing that ‘cities, as the physical objects we generally envision them to be, are also cognitive objects, that is, they are not something only out there but also a type of extensions of the human mind.’ As noted above, such ‘extended cognition’ follows naturally from a dynamical systems understanding of affordances (Chemero, 2003, 2009; Rockwell, 2005; Anderson et al., 2012).

The transactional nature of affordances suggests that, when designing urban environments, local attitudes and interests should also be charted in an inclusive and even participatory process. In other words, affordances “cannot be imposed by expertise themselves but need to consider the ‘meanings’ of the local community” and “cannot be implemented as abstract ‘demands’ but have to cognitively engage and motivate people, even if on a low key” (Marcus et al., 2016, p. 443). Kurz (2002, p. 273) supports this idea by noting that financial rebates on public transport systems are not sufficient if, for example, people are more attuned to the social status their private vehicles afford them with. Interestingly, Kurz’s notions on public transport are supported by a study by Hunecke et al. (2001), which suggests that an additive ‘economy-plus-moral’ (subway fare plus normative ecological orientation, i.e., external plus internal) formula best determines public transport travel choice in urban environments. As discussed extensively above, environments do not afford pro-environmental behavior alone, but always in relation to human abilities and motivations.

Marcus et al. (2016, p. 446) also cite their previous work (Giusti et al., 2014) to highlight the importance of green urban affordances. The provision of green affordances (accessibility to urban nature in Stockholm) for preschool children was shown to lead to increased ecological knowledge and impact awareness, as well as strengthened emotional connection with nature. These internal factors, again, could be termed as further latent potential for pro-environmental behavior. In this respect, urban environments can ratchet cognitive processes: by redesigning urban environments to reinforce sustainable affordances (e.g., accessibility to nature), we can promote a wealth of pro-environmental identities and habits, which may over time reinforce the transition toward a culture of sustainability.

In fact, the notion of the affordance could be extended to include a whole socioeconomic system. This goes far beyond the scope of this article, but (e.g., Sen’s, 1995) capability approach has elaborated a very similar idea, where abstractions such as ‘equality’ and ‘freedom’ are assessed as the actual capabilities (which relate to both individual physical abilities, or ‘functionings’, and the system’s distribution of action opportunities) of human-beings. In other words, even concepts such as freedom, justice, equality can be assessed in terms of functionally meaningful human–environment relations (where the environment, of course, includes social, cultural and economic determinants).

## Conclusion

‘There is only one world, however, diverse, and all animals live in it, although we human animals have altered it to suit ourselves. We have done so wastefully, thoughtlessly, and, if we do not mend our ways, fatally’ (Gibson, 1979, p. 130).

Affordance theory, as presented in this paper, studies the dynamics of organism–environment systems and their evolution over time. In this context, internal and external behavior antecedents should not only be studied as interdependent entities, but as a single, non-decomposable, evolutionary system (**Figure 1**). In this respect, affordance theory particularly helps us understand the ecology of environmentally significant behavior. It has been proposed in this paper that those involved with environmental policies adopt affordance theory as a guiding heuristic for the design and implementation of pro-environmental behavior change interventions. Several reasons exist to as why this should be the case:

(1) Affordances can be understood to represent leverage points for systemic behavior change interventions, since their actualization can lead to large and self-reinforcing shifts in environmentally significant behavior. Affordance theory, as a dynamical systems approach, can therefore guide us to conceptualize and identify leverage points which can help individuals and socio–ecological systems to learn more persistent sustainable habits. This second-order change (helping the system learn) not only solves particular individual problems, but also forms habits which apply to the solution of classes of problems (Bateson, 2000, p. 274).
(1) By identifying and activating pro-environmentally significant affordances in large enough numbers, we can induce positive feedback loops (**Figure 1**), where, for instance, changes in the material environment reinforce pro-environmental identities and promote pro-environmental sociocultural practices (which again can lead to further modulation of the socio-material environment, and so on). The reinforcement of these feedback loops can further serve to normalize pro-environmental habits as socio-culturally and materially embodied practices.
(1) Affordance theory, as an ecological approach to behavior analysis, helps us conceptualize and understand the lack of fit between internal and external behavior antecedents. Focus in policy interventions should be particularly directed to domains where mismatches between internal and external factors exist. One exemplary case is the widely prevalent attitude–action gap, where latent pro-environmental internal factors pre-exist but are not yet actualized due to lack of affordances. The actualization of pre-existing pro-environmental internal factors is also more likely to lead to positive spillover effects than other interventions (Truelove et al., 2014), making it a particularly important leverage point.
(1) Affordance theory is a particularly useful and versatile conceptual framework for policy interventions, since it is, due to its systemic and nested nature, applicable to practically any environmentally relevant policy-arena from the sustainable design of objects and households to urban environments.

This, I believe, presents us with a conceptual framework for a systemic *mending of our ways* (in reference to Gibson above) toward a sustainable future, where pro-environmental behavior would emerge as a default path of least resistance and form of life. Moreover, affordance theory has the potential to be a participatory approach at that. A thorough understanding of latent local behavior potential seems to call for participatory and decentralized policy-making, with heightened understanding of locally embedded meanings and local environments. This also has the potential to increase the social and political acceptability of behavior change interventions (which ‘nudge’ interventions, in particular, have struggled with, see e.g., Hukkinen, 2016) and reduce helplessness in local populations. Whilst this mending of our ways is by no means an easy task (and much is left to be studied in how pro-environmental feedback loops can be practically implemented) and not perhaps the radical systemic change some commenters seem to call for (see e.g., Capstick et al., 2015), there are reasons to be optimistic that little strokes fell great oaks. After all, any organism–environment system is necessarily infused with affordances, and by mending affordances toward a self-reinforcing, less wasteful and thoughtless, direction there is hope that our socio–ecological system will ultimately take a sustainable turn.

## Author Contributions

RK made sole contribution to the conception and design of the work. RK drafted the work and revised it critically for important intellectual content. RK agrees to be accountable for all aspects of the work in ensuring that questions related to the accuracy or integrity of any part of the work are appropriately investigated and resolved.

## Conflict of Interest Statement

The author declares that the research was conducted in the absence of any commercial or financial relationships that could be construed as a potential conflict of interest.

## Footnotes

1 I acknowledge here, at the very beginning of this text, that the use of the terms ‘internal’ and ‘external’ imply a dichotomy that, ontologically speaking, simply does not exist (it is a ‘false dichotomy’; Gibson, 1979). Indeed, this paper is devoted to show that they are not dichotomous, and are instead mutually implicative and systemically relative (there is no internal without external). However, due to linguistic insufficiencies I maintain that a cautious upholding of this dichotomy is necessary for pragmatic purposes.

2 The transitive verb *to afford*, in the whole of this text, should be read to imply the meaning ‘to make available, give forth, or provide naturally or inevitably’ (as defined by the Merriam-Webster dictionary). This should not be confused with the more common definition ‘to be able to bear the cost of’ something. The noun *affordance* is a neologism coined by ecological psychologist James J. Gibson (see e.g., Gibson, 1979) and, of course, refers merely to the former definition of the verb ‘to afford.’ To cite Gibson (1979, p. 127) himself, ‘The verb *to afford* is found in the dictionary, but the noun *affordance* is not. I have made it up. I mean by it something that refers to both the environment and the animal in a way that no existing term does. It implies the complementarity of the animal and the environment.’

3 I suggest that affordances are best interpreted via an anti-Cartesian ontology, which does not separate subjects from objects. Good candidates for such an ontology can be found in early American pragmatism, for instance the works of John Dewey (e.g., Dewey, 1958) and William James (see Heft, 2001). This is no coincidence, since Harry Heft’s (Heft, 2001) brilliant exposition on ecological psychology reveals James’ radical empiricism’s direct influence on James J. Gibson’s ecological psychology. Common to these perspectives is the ontological priority of *processes* over *substances*, sometimes labeled ‘process metaphysics’ or ‘process philosophy’ (Rescher, 1996, 2000). For an introduction to process philosophy see the work of Nicholas Rescher (Rescher, 1996, 2000), and for an interpretation of process philosophy in the context of environmental policy, see Kaaronen (in press).

4 Interestingly, Polanyi’s philosophy of tacit knowing seems to have, to some extent, influenced Gibson (1979, 22, 260–261). Drawing on Polanyi, Gibson notes that knowledge can be said to be both *tacit* and *explicit*, but that there has to be a tacit ‘awareness of the world’ before it can be explicated (‘put into words’); ‘perceiving precedes predicating’. Gibson continues: ‘However, skilled an explicator one may become one will always, I believe, see more than one can say’. See also Kaaronen (in press) for discussion on sustainability, M. Polanyi and affordances.

5 In fairness, it should be acknowledged that cognitive dissonance could, at best, result in Alfa and Beta going through excess measures to recycle (i.e., adjust behavior to match values), although it is arguably more likely that they take the ‘path of least resistance’. As Cooper (2007, 8) notes, ‘the relative ease of changing one’s attitudes rather than one’s behavior has made dissonance more relevant to attitudes than to any other concept.’

6 This is similar to the concept ‘virtuous circle’ (or ‘foot-in-the-door’) in social psychology. For example, children who perceive themselves as being generous because of a previous act of (even haphazard) generosity are more likely to continue to behave generously (Tavris and Aronson, 2015). This is predicted by the theory of cognitive dissonance: when someone behaves in a certain manner, they are likely to afterward self-justify the previous behavior in order to maintain consonance (Festinger, 1957). Sustainable behavior can lead to sustainable thinking (and vice versa) in a ‘virtuous circle’ or sustainable feedback loop.

7 A complicating factor here is that it seems that high-cost PEB is more likely to promote positive spillover PEB than low-cost behavior (Truelove et al., 2014). This makes sense from a cognitive dissonance perspective: when a person is highly invested in one practice, they are likely to self-justify other similar behavior (Cooper, 2007; Tavris and Aronson, 2015). The question for policy-makers remains: how can people be supported to act in consonance with action they perceive as high-cost?

8 From other theoretical frameworks possibly relatable to affordance theory, I can think of at least Giddens (1984) structuration theory, Lewin’s (1951) field theory, Sen’s (1995) capability approach, practice theory (e.g., Bourdieu, 1990 and Shove et al., 2012), Paul Stern and colleague’s (Guagnano et al., 1995; Stern, 2000) attitude–behavior–context model, nudge theory from behavioral economics (Thaler and Sunstein, 2008) as well as the whole discourse on ecosystem services (see e.g., Danley and Widmark, 2016).

9 The Heideggerian notion of ready-to-handedness (see Heidegger, 1927/1978) is often implicit in ecological psychology (see e.g., Chemero, 2013). Being ‘ready-to-hand,’ very briefly, implies that an object ‘is for’ (or affords) the achieving of some function without the need for theorizing or other analytical activities. For those familiar with behavioral economics, particularly nudge theory (Thaler and Sunstein, 2008) and Kahneman’s (2011) dual system approach, there is something inherently System 1 (fast, automatic behavioral processes) in ready-to-handedness.
